# Trichobezoar-Induced Heartburn in a Teenage Girl: A Case Report

**DOI:** 10.1177/2324709618776345

**Published:** 2018-05-18

**Authors:** Shahrazad Akbar, Lujayn Akbar, Nuralhuda Akbar, Ali Nawras

**Affiliations:** 1 King Fahd Hospital of the University, AL Khobar, Saudi Arabia; 2 University of Toledo Medical center, Toledo, OH, USA

**Keywords:** trichobezoar, heartburn, trichotillomania, trichophagia, abdomen, bezoars, gastric outlet obstruction, abdominal pain

## Abstract

One of the most commonly encountered medical problems affecting all age groups in health care is abdominal pain. There are many surgical and medical causes behind this symptom; however, a rare cause of abdominal pain and other unspecific abdominal complaints are gastric bezoars. Gastric bezoars are defined as the accumulation of undigested or partially digested foreign materials in the stomach. They are typically found incidentally during upper endoscopy. Patients may present with abnormal behavior or eating disorders such as pica. Therefore, proper history taking and establishing a good rapport with the patient play a key role in diagnosis. We present a case of trichobezoar-induced heartburn in an 18-year-old female. In this article, we will discuss the types, risk factors, clinical picture, diagnosis, and treatment of this condition.

## Introduction

Gastric bezoars are defined as the accumulation of undigested or partially digested foreign materials in the stomach. Bezoars are termed trichobezoar when they are composed of hair.^1^ The word trichobezoar originates from the word “bazahr” used by Arab medical writers to describe poison antidotes extracted from an animal’s abdomen, and the Greek word for hair, “trich.” The condition is often associated with trichotillomania (obsessive hair pulling) and/or trichophagia (hair swallowing). Bezoars are a rare cause of abdominal pain and other unspecific abdominal complaints such as heartburn, constipation, and anorexia. Therefore, thorough history taking in cases with unspecific gastrointestinal symptoms in the absence of a probable underlying cause should include inquiring about excessive ingestion of such materials.

## Case Presentation

This is a case of an 18-year-old Caucasian female who was referred to us from an outlying facility for evaluation of heartburn and chronic abdominal pain. The patient started experiencing the pain several months before presentation. The pain was cramping in nature, intermittent, occurring approximately 3 times per week, lasting for a few hours with no relieving or aggravating factors. There was no history of altered bowel habits. The patient gave no history of previous surgeries. On further questioning, a positive history of hair eating (trichophagia) was obtained but with no history of alopecia, eating pattern change, thoughts of body image distortion, or previous psychiatric disorders.

General examination of the patient was unremarkable with no signs of dehydration, halitosis, or lymphadenopathy. Local abdominal examination revealed diffuse mild tenderness of the abdomen. No palpable masses were appreciated. Normal bowel sounds were present on auscultation.

**Figure 1.**
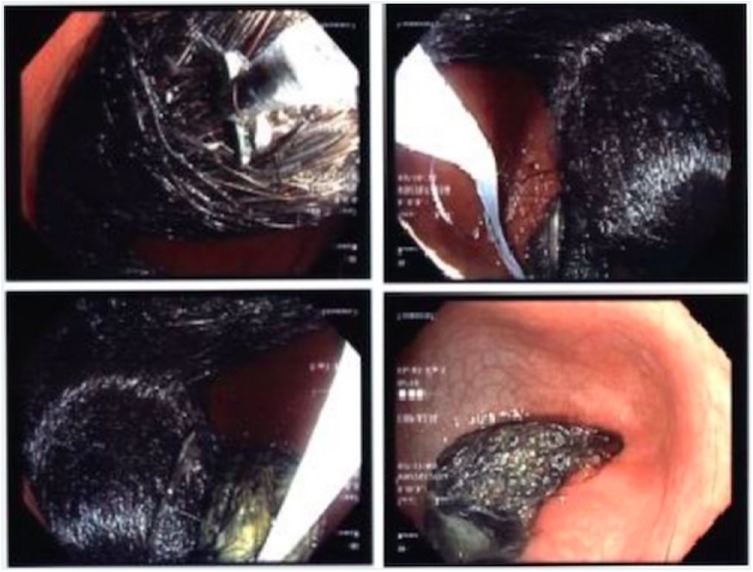
Endoscopy showing gastric trichobezoar in the stomach.

Laboratory investigations detected microscopic anemia and mild leukocytosis. An upper gastrointestinal X-ray with air contrast was performed at an outlying facility and showed a distended stomach that appeared to be filled with debris, along with a normally filled duodenum. Esophagogastroduodenoscopy was performed at our institution, which demonstrated a trichobezoar occupying 75% of the gastric lumen extending down through the pylorus and into the duodenal bulb (Figure 1).

A few attempts to remove the hair with scissors and forceps were made, but none were successful. Exploratory laparotomy and gastrostomy were performed afterward in which the bezoar was removed successfully and without any complications. It measured 17 × 6 × 7 cm and weighed 2.33 lbs. The patient recovered well and was discharged home with psychiatry clinic follow-up.

## Discussion

Heartburn and abdominal pain are common medical complaints seen in all ages including teenagers. History is helpful in identifying the underlying cause. Every so often, however, a case may present itself with a history that does not point to a favorable diagnosis; such is the case with gastric trichobezoars. It is a rare medical condition with clinicians encountering it around 0.3% of performed upper gastrointestinal endoscopies.^1^

A gastric bezoar is defined as the accumulation of undigested or partially digested foreign materials in the stomach. Bezoars are classified based on their composition and/or location. Materials that compose bezoars include but are not limited to vegetable matter (phytobezoars), which are the most common type of bezoars, persimmon fruit (diospyrobezoars), which are the most common subtype of phytobezoars, hair (trichobezoars), and ingested medications (pharmacobezoar), for example, enteric-coated aspirin, nifedipine, theophylline, and sodium alginate. Other materials that can form bezoars include fungi, cement, milk curd, tissue paper, and vinyl gloves.^1-7^

The process of formation of trichobezoar begins with hair accumulation in the gastric folds. Human hair by nature is indigestible and resists peristalsis. Hair proteins get denatured by gastric acid and become oxidized, which in turn causes the change of hair color to black, regardless of the original hair color.^1,8^ Eventually, bezoars will grow with the accumulation of food, especially cellulose-rich foods and other indigestible materials, and form a conglomerate of various materials. Bacteria may colonize this collection resulting in halitosis.^1^ Hair accumulation occurs in the stomach but can also extend to the intestines, which may lead to Rapunzel syndrome.^9^

Previously, it was thought that bezoars were formed due to delayed gastric emptying. However, studies have shown that patients with normal gastric emptying can also develop them.^8^ People at a higher risk for developing bezoars are those with altered gastrointestinal motility or anatomy such as diabetics, patients with neurological disorders, or patients with previous gastric surgeries. Psychiatric illnesses and mental retardation are associated with bezoars due to pica, compulsive and excessive ingestion of food, or inedible substances. Trichobezoars, in particular, are more prone to develop in females and adolescents.^1,8,10^

Patients may remain asymptomatic for extended periods of time. If symptomatic, typical clinical presentation includes upper abdominal pain, nausea, vomiting, weight loss, anorexia, early satiety, and symptoms of anemia such as dizziness, palpitations, and easy fatigability.^1,2,8^

Clinical examination is usually unremarkable. Occasionally, an abdominal mass may be appreciated on palpation. Halitosis may also be present. Alopecia characterized by areas of patchy hair loss with broken hair strands of variable lengths indicate an association with trichotillomania and trichophagia.^1,8^

Diagnosis is made using radiological studies such as abdominal X-ray with or without contrast and computed tomography scan or ultrasonography that reveal calcified, granular, or swirl-like structures of solid and gaseous material or filling defects within the stomach. Computed tomography with an orally administered contrast may reveal free-floating filling defects. It is necessary to exercise caution when diagnosing trichobezoars as they may easily be mistaken for malignancies of the gastrointestinal tract. On upper gastrointestinal endoscopy, trichobezoars appear as a black, green, or brown mass found in the funds or antrum of the stomach. Effort should be made to search for coexisting gastric ulcers, perforation, or any other gastric pathology.^1,2,8^

A gold standard treatment plan is controversial due to the lack of studies that compare the different treatment modalities. Chemical dissolution of the mass can be used to treat patients with mild symptoms. Patients with two failed trials at chemical dissolution, resistant materials to chemical dissolution such as trichobezoar or moderate to severe symptoms should undergo upper endoscopy and have the mass endoscopically removed. The trichobezoar, however, may break and fragments may get displaced into the small bowel and cause obstruction. Surgery is reserved for cases with failure of the aforementioned treatment methods or complicated trichobezoars. Laparotomy with gastrotomy is the usual method of choice in such patients.^1,8^

Bezoars may be complicated by pancreatitis, obstructive jaundice, steatorrhea, gastrointestinal perforation, peritonitis, protein-losing enteropathy, intussusception, appendicitis, constipation, pneumatosis intestinalis, and rarely gastric outlet obstruction.^9,11,12^

Prognosis after removal is good. Recurrence can occur in 20% of patients and can be prevented by psychiatric evaluation, controlling precipitating habits, increasing water consumption, and assessment of possible underlying causes.^1,8^

## Conclusion

Gastric trichobezoar, a bezoar composed of hair in the stomach, could be the cause of chronic abdominal pain or heartburn. It should be suspected when a likely underlying cause is not established particularly in patients with psychiatric disorders or with a history of trichotillomania and trichophagia. Good history taking plays a key role in diagnosing such cases. Diagnosis depends on upper gastrointestinal endoscopy. A gold standard treatment plan is controversial due to the lack of studies that compare the different treatment modalities. After successful management, psychiatric evaluation should be considered and the patient should be educated to prevent recurrence.
